# The brainstem in multiple sclerosis: MR identification of tracts and nuclei damage

**DOI:** 10.1186/s13244-021-01101-7

**Published:** 2021-10-21

**Authors:** Thien Huong Nguyen, Alexis Vaussy, Violette Le Gaudu, Jennifer Aboab, Sophie Espinoza, Irina Curajos, Emmanuel Heron, Christophe Habas

**Affiliations:** 1 Department of Neuro Imaging, C.H.N.O. des Quinze- Vingts, Paris, France; 2 Siemens Healthcare France, Saint-Denis, France; 3 Department of Internal Medicine, C.H.N.O. des Quinze-Vingts, Paris, France

**Keywords:** MRI, Multiple Sclerosis, Brain stem, Anatomy

## Abstract

**Objective:**

To evaluate the 3D Fast Gray Acquisition T1 Inversion Recovery (FGATIR) sequence for MRI identification of brainstem tracts and nuclei damage in multiple sclerosis (MS) patients.

**Methods:**

From april to december 2020, 10 healthy volunteers and 50 patients with remitted-relapsing MS (58% female, mean age 36) underwent MR imaging in the Neuro-imaging department of the C.H.N.O. des Quinze-Vingts, Paris, France. MRI was achieved on a 3 T system (MAGNETOM Skyra) using a 64-channel coil. 3D FGATIR sequence was first performed on healthy volunteers to classify macroscopically identifiable brainstem structures. Then, FGATIR was assessed in MS patients to locate brainstem lesions detected with Proton Density/T2w (PD/T2w) sequence.

**Results:**

In healthy volunteers, FGATIR allowed a precise visualization of tracts and nuclei according to their myelin density. Including FGATIR in MR follow-up of MS patients helped to identify structures frequently involved in the inflammatory process. Most damaged tracts were the superior cerebellar peduncle and the transverse fibers of the pons. Most frequently affected nuclei were the vestibular nuclei, the trigeminal tract, the facial nerve and the solitary tract.

**Conclusion:**

Combination of FGATIR and PD/T2w sequences opened prospects to define MS elective injury in brainstem tracts and nuclei, with particular lesion features suggesting variations of the inflammatory process within brainstem structures. In a further study, hypersignal quantification and microstructure information should be evaluated using relaxometry and diffusion tractography. Technical improvements would bring novel parameters to train an artificial neural network for accurate automated labeling of MS lesions within the brainstem.

## Key points

- Assessment of elective brainstem tracts and nuclei affected by MS using FGATIR and PD/T2w sequences.
- Brainstem structures identification using a dedicated high resolution FGATIR sequence.
- FGATIR provides small structures delineation for oncoming tractography evaluation in patients affected by MS.

## Introduction

Multiple sclerosis (MS) is an inflammatory disease of the central nervous system (CNS). It is the most disabling non-traumatic disease in young people, affecting 2 to 3 times more women than men. Most of the times, neurological deficits appear between 20 and 40 years of age, with variable clinical courses [1–3].

At the junction between the brain and the spinal cord, the brainstem represents a complex interdigitation of compact anatomic pathways and nuclei surrounded by the reticular formation. Although MS diagnosis in this essential structure seems crucial to predict long term disabilities, so far few studies have explored the spatial distribution of MS lesions within the brainstem [4]. Usual conventional MRI sequences fail to discriminate thoroughly the nuclei and tracts composing the brainstem because of their small volume [5–9]. Therefore, radiologists are compelled to infere the location of these small structures using anatomical atlases derived from animal histological studies [10–12].

Initially developed for deep brain stimulator implantation by Sudhyadom et al., the Fast Gray Matter Acquisition T1 Inversion Recovery (FGATIR) has been proposed as a modification of the standard Magnetization Prepared Rapid Acquisition Gradient Echo (MPRAGE) sequence, characterized by a shorter inversion time that specifically suppresses the white matter signal [13, 14]. Since FGATIR provides an increased contrast between the gray matter and the signal suppressed white matter, a high spatial resolution and single-millimeter slice visualization, FGATIR appears particularly suitable for small brainstem structure discrimination. Recently, Shepherd et al. (2020) delineated nuclei and tracts within the brainstem, using FGATIR with a 1 mm isotropic resolution [15]. Despite a long acquisition time of 12 min, the authors concluded that FGATIR may be highly informative in the initial diagnosis and follow up of inflammatory processes of the CNS.

In this work, we aimed to evaluate the efficiency of a dedicated high spatial resolution FGATIR to localize MS lesions within the brainstem.

## Material and methods

### Study population

This study was an observational study performed retrospectively and approved by our institutional review board and ethics committee in November 2020. From April to December 2020, 50 patients who were addressed to our institute for MRI follow up with relapsing remittent MS were included: 29 women, 21 men (sex ratio 1.38), aged from 22 to 69 years at the time of imaging (median age 40.6 years). Ten age matched control subjects without MS history were recruited for comparative imaging during the same period. All patients and controls subjects were informed and gave written consents. The data supporting this study are available from the corresponding author on reasonable request.

### MR protocol

MR imaging was performed on a 3 T system (MAGNETOM Skyra, Siemens Healthcare) using a 64-channel head coil. A 3D FGATIR sequence was performed on the 10 healthy patients to identify macroscopic structures of the brainstem such as tracts and nuclear groups. The FGATIR sequence was performed with the following acquisitions parameters: TR/TE/TI = 3000/3.59/403 ms, FOV = 242 × 256 mm, base resolution = 272 × 288, bandwidth = 220 Hz/Px, 3 excitations. FGATIR was acquired in axial orientation to maximize the in-plane resolution in correlation with other conventional MRI sequences. To provide with a clinical compatible scan time of 14 min 42 s, the slab thickness was interpolated to 1.6 mm and reconstructed at 0.9 mm.

In MS patients, the FGATIR sequence was added to the usual MRI exploration protocol which included a 3D T1 MPRAGE, a 3D FLAIR, a 3D T1 post contrast imaging as well as a 2D Proton Density/T2 weighted dual echo (PD/T2w).

The 3D T1 MPRAGE sequence was acquired with the following parameters: TR/TE/TI = 2200/3.6/900 ms, FOV = 256 × 256 mm, base resolution = 256 × 256, slice thickness = 1 mm, bandwidth = 240 Hz/Px, one excitation, scan time = 3 min 22.

The 3D FLAIR sequence was acquired with the following parameters: TR/TE/TI = 7000/380/2225 ms, FOV = 256 × 256 mm, base resolution = 230 × 256 mm, slice thickness = 1.3 mm, bandwidth = 814 Hz/Px, two excitations, scan time = 4 min 47.

The 3D T1 TSE post contrast imaging sequence was acquired with the following parameters: TR/TE = 700/19 ms, FOV = 230 × 230 mm, base resolution = 256 × 256 mm, slice thickness = 0.9 mm, bandwidth = 751 Hz/Px, two excitations, scan time = 3 min 28.

The 2D Proton Density/T2 weighted dual echo (PD/T2w) sequence was acquired with the following parameters: TR/TE = 4000/27/95 ms, FOV = 203 × 240 mm, matrix = 292 × 384, slice thickness = 3 mm, bandwidth = 310 Hz/Px, one excitation., scan time = 3 min 12.

The total MRI protocol duration was 29 min 03.

### Image analysis

#### Anatomical structures identification

Structure identification was performed by 2 neuro-radiologists (T.H.N. and V.L.G. with respective experience of more than 20 years and 4 years in neuroradiology) using standard anatomical references and nomenclature.

As a first step, an anatomical labeling was performed in control subjects. This learning step allowed the reader to perform brainstem tracts and nuclei identification based on the distinctive FGATIR signal of the brainstem structures. 3D reconstructions of macroscopic distinctive structures were correlated to anatomical atlases and histological sections [11, 16–19]. Applying FGATIR sequence onto the 10 controls subjects aimed to assess the consistency of the image quality and to allow anatomical identification of all subjects examined.

#### MS hypersignals assessment

As a second step, the same FGATIR sequence was applied in MS patients. Hypersignal lesions were first identified on PD/T2w axial sections [20, 21]. Inclusion criteria of a brainstem hypersignal included: (i) a hypersignal across two contiguous slices or (ii) a hypersignal identified on a single slice with a minimum surface size of 1.25 mm^2^, corresponding to two adjacent pixels.

For unilateral structures affected by MS, further quantitative evaluation were performed in patients, who were used as their own control. Using a syngo.via workstation (Siemens Healthcare, Erlangen), identical regions of interest (ROIs) were drawn in apparent hypersignals and normal contralateral structures on PD/T2w. An additional ROI was drawn on the cerebrospinal fluid as reference. Signal intensity measurements (SI) were focused on structures frequently involved in MS, such as the medial longitudinal fasciculus, the trigeminal tract, as well as corticospinal pyramids [22–27].

The lesion contrast ratio (CR_Lesion_) was calculated as a measure of the lesion conspicuity (SI_Lesion_) against CSF background (SI_CSF_), normalized to the CSF standard deviation (SD_CSF_). The CSF areas were chosen in the same slices in which the lesions and contralateral structures signal intensity were measured.

Contrast Ratio (CR) was calculated according to the following formula

$${\text{CR}}_{{{\text{Lesion}}}} = ([{\text{SI}}_{{{\text{Lesion}}}} - {\text{SI}}_{{{\text{CSF}}}} {\text{l}}]/{\text{SD}}_{{{\text{CSFl}}}} )$$

A similar ratio was calculated for normal appearing contralateral structure (CR_Contralateral_):

$${\text{CR}}_{{{\text{Contralateral}}}} = \left( {\left[ {{\text{SI}}_{{{\text{Contralateral}}}} - {\text{SI}}_{{{\text{CSF}}}} {\text{l}}} \right]/{\text{SD}}_{{{\text{CSF}}}} } \right)$$

Finally, hypersignals identified with PD/T2w were overlayed onto the anatomical structures identified with FGATIR to evaluate the hypersignal incidence frequency on macroscopically individualizable structures.

### Statistical analysis

Data were analyzed using R software v3.3.2 (The R Foundation, Inc., USA). Quantitative variables were described using mean and standard deviation (mean ± SD) and qualitative variables with numbers and percentages. CR_Lesion_ and CR_Controlatateral_ were compared using a non-parametric statistical test (Wilcoxon signed rank test). A statistical evaluation compared the interobserver agreement between the 2 radiologists for MS hypersignal localization through PD/T2w and FGATIR sequences, using Cohen’s kappa test. General rules for the interpretation of Cohen's κ coefficient were used, i.e. < 0 = poor agreement, 0.01–0.20 = slight agreement, 0.21–0.40 = fair agreement, 0.41–0.60 = moderate agreement, 0.61–0.80 = substantial agreement and 0.81–0.99 = almost perfect agreement.

Statistical significance was set for a *p* value < 0.05.

## Results

### Anatomical identification of normal structures of the brainstem

**Fig. 1:**
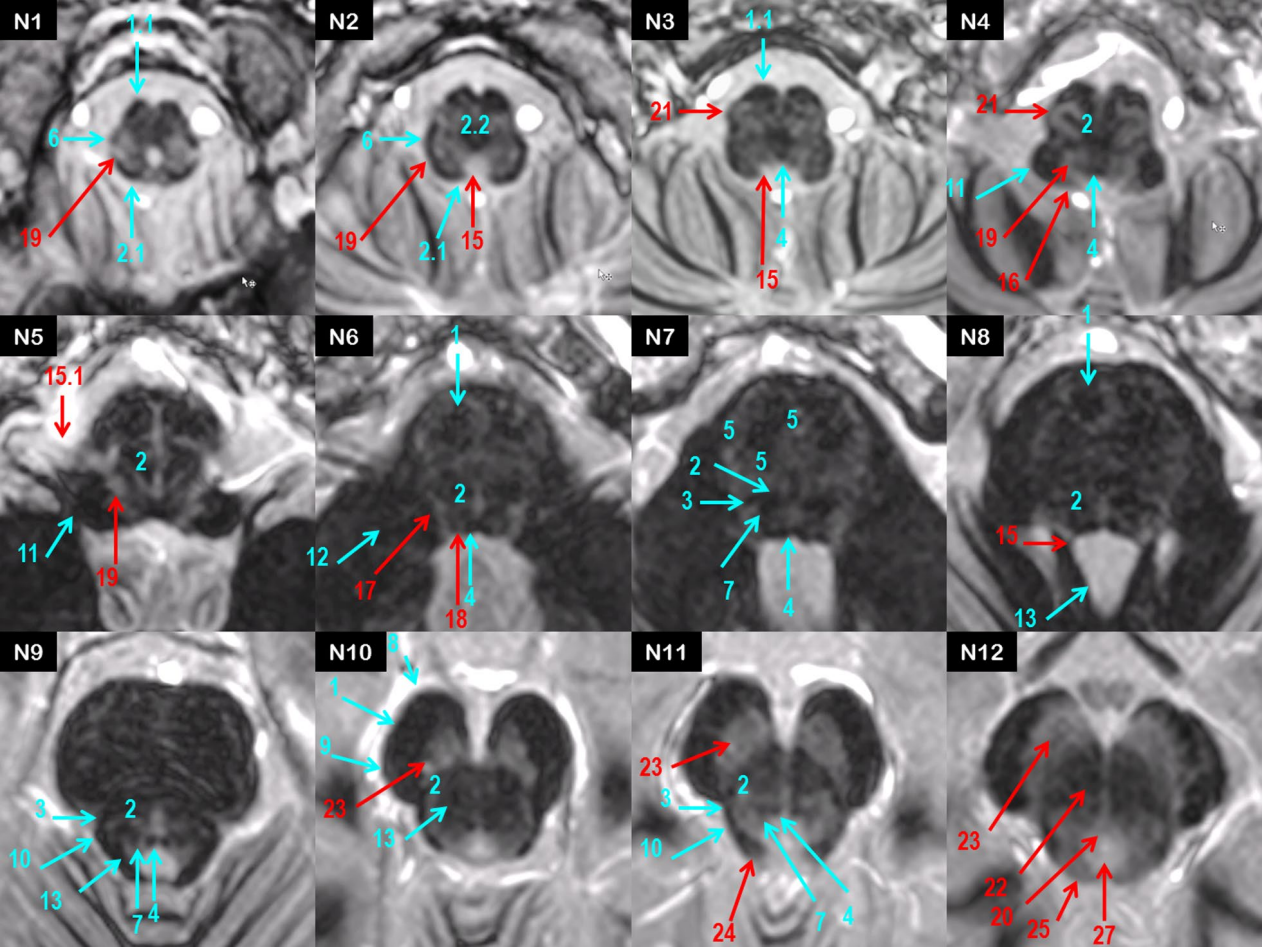
Inferior-to-superior axial MR FGATIR representation of normal brainstem structures from N1 to N12 (tracts in cyan, nuclei in red)

Within the normal brainstem, the most hyperintense structure was the periventricular gray matter such as the midbrain periaqueductal gray [15]. Other hyperintense structures were central gray matter such as the substantia nigra and pontine nuclei. Structure signal decreased inversely to myelin content, as for the nuclei of cranial nerves, the inferior and superior colliculi and the reticular formation, successively. Myelinated brainstem pathways were identified as the most hypointense structures. The spatial resolution of FGATIR offered a precise and reproducible identification of several macroscopic entities from the caudal medulla oblongata to the rostral midbrain, as shown in Fig. 1.

At the spinal cord–medulla oblongata junction (N1–N4), afferent and efferent tracts appeared as hypointense structures: the cortico spinal tract pyramids (CST, 1.1), the spino cerebellar tract (SCT, 6) bordering laterally the hyperintense trigeminal tract (V, 19). The dorsal paramedial gracile and cuneate fasciculi (GFCF, 2.1) gathered in internal arcuate fibers (IAF, 2.2) before crossing the midline into the medial lemniscus (ML, 2). From N3 to N5 were successively visualized in moderate hypersignal the solitary nucleus and the solitary tract (15) resulting in the vague nerve (15.1).

At the pons level (N5-N9), ML (2) appeared as a transversal hypointense tract, separating the tegmentum pontis from the basis pontis where crossed the transversal fibers of the pons (TFP, 5) and CST (1). ML also provided identification of neighboring small tracts such as the spino thalamic tract (STT, 3), the central tegmental tract (CTT, 7) coming from the olivary complex (OC, 21). In the caudal tegmentum pontis, the hypointense dot of the medial longitudinal fasciculus (MLF, 4) was bordered by the abducens nucleus (VI, 18) which was surrounded by the genu of the facial nerve (VII, 17) in the paramedial bulgy eminentia teres. Laterally were located the nuclei of VII and V in caudal pons (17) and rostral pons (19), respectively.

At the midbrain level (N10-N12), the peri acqueductal gray (PAG) gave a large hyperintense ring surrounding the aqueduct (27), ventrally borded by the III nucleus (20, N12) at the superior colliculus level (25). ML was laterally shifted by the decussation of the superior cerebellar peduncle (SCP, 13) and the red nucleus (RN, 22). At the hinge between the tegmentum and the crus cerebri, STT (3) appeared as a hyposignal at the angle formed medially by ML and dorsally by the lateral lemniscus (LL, 10), the latter ending up at the inferior colliculus (24) with a classical “golf tee” shape. The substantia nigra (23) was identified as a large hyperintensity delimited by the efferent tracts of the crus cerebri, from medial to lateral the frontopontine fibers (FPF, 8), the corticobulbar and corticospinal fibers (1), and the parietopontine, occipitopontine, and temporopontine fibers (POTPT, 9). The inferior, middle and superior cerebellar peduncles gave lateral borders to each level of the brainstem (11, 12, 13). Running through the brainstem, both MLF tracts were easily discernable as a hypointense double dot at the periventricular midline in axial sections (4).

### Brainstem involvement in MS

**Fig. 2:**
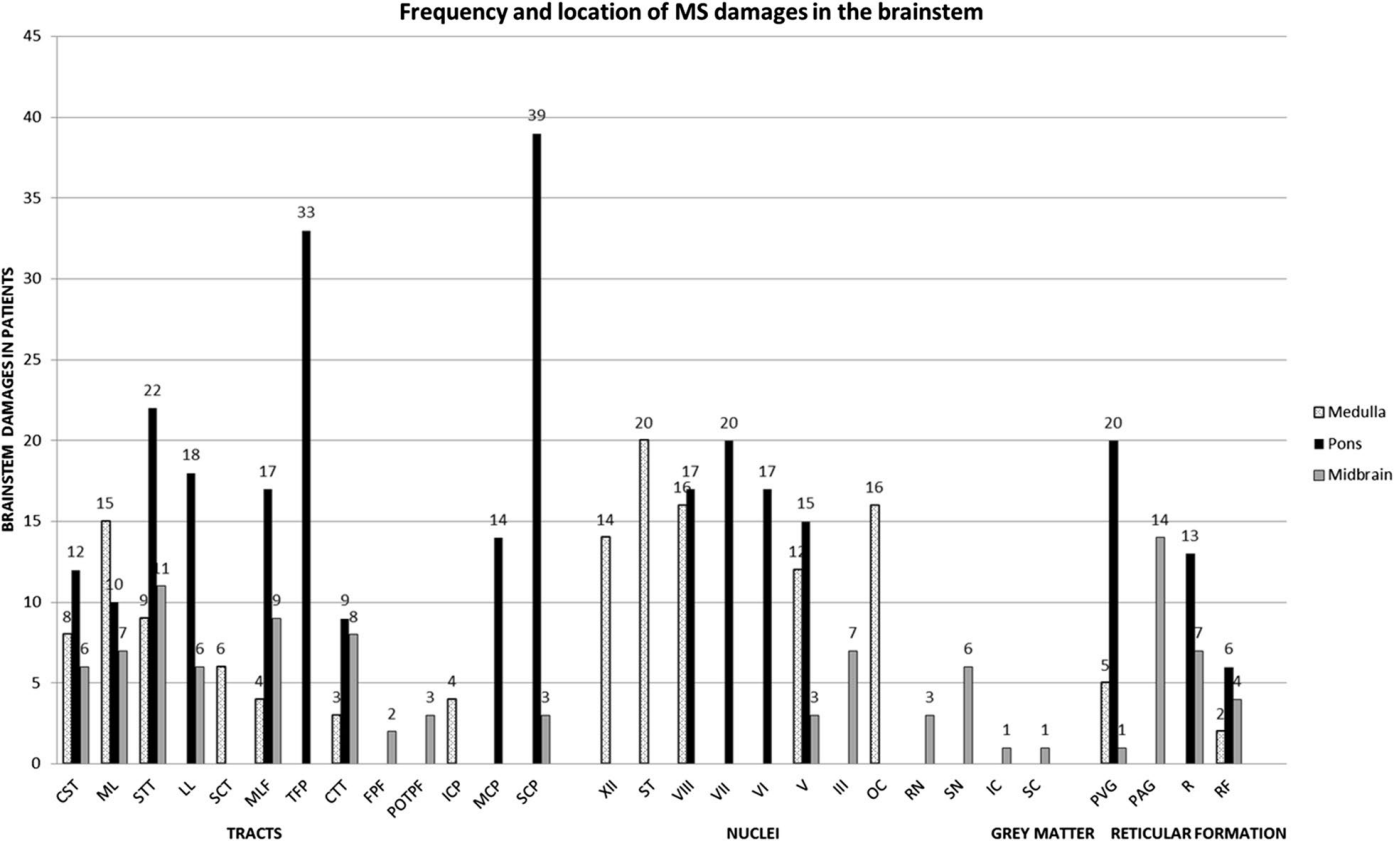
Frequency and location of MS damages in the brainstem

**Fig. 3:**
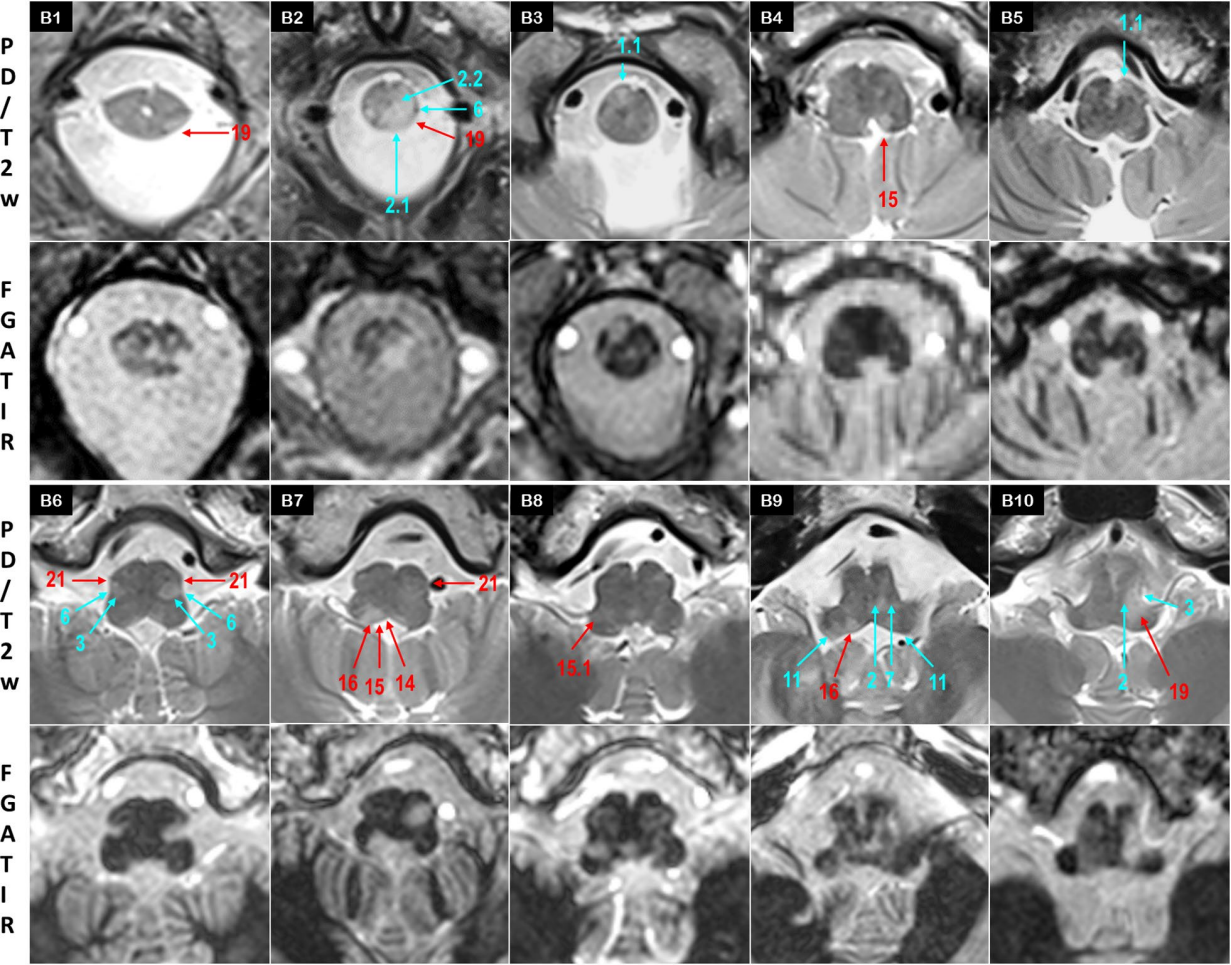
MS identification at the medulla oblongata, caudal level (B1–B5) and rostral level (B6–B10)

**Fig. 4:**
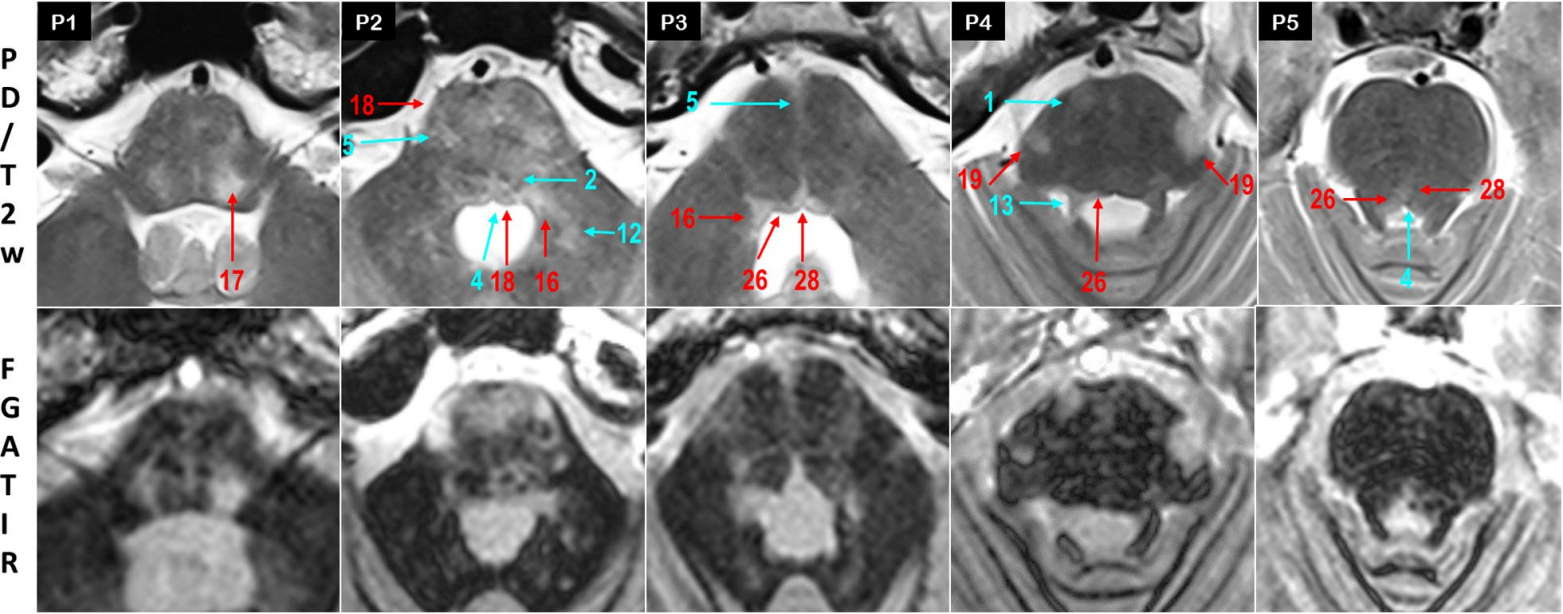
MS identification at the pons level

**Fig. 5:**
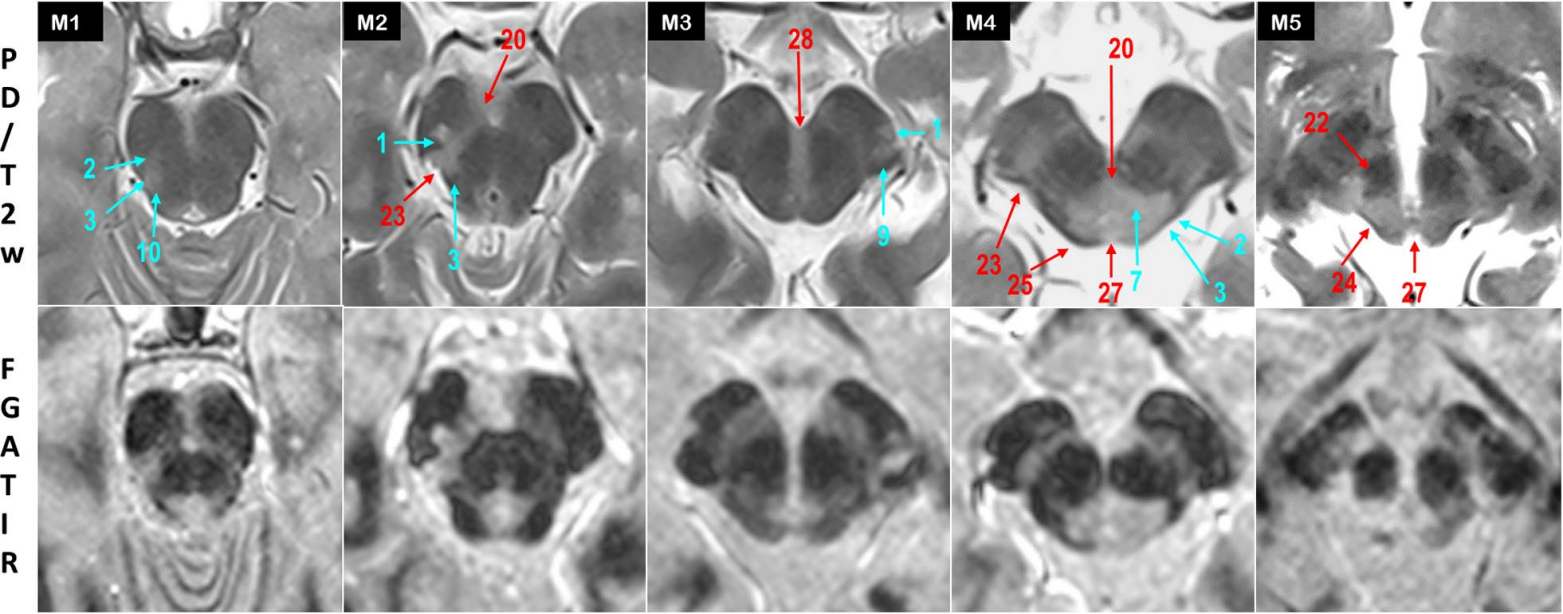
MS identification at the midbrain level

The Table 1 and Fig. 2 indicate the frequency and location of brainstem damages for the 50 MS patients. Illustrations of MS brainstem involvement are proposed on Figs. 3, 4 and 5. While most identifiable nuclei were delineated on a specific area (except for the extended V tract), hypersignals in afferent and efferent tracts might be found at different levels of the brainstem. For this reason, tracts affected by MS were identified according to their location on medulla, pons and midbrain, and bilateral lesions were considered as one lesion.

**Table 1:** Frequency and location of MS damages in the brainstem

Structure	Medulla	Pons	Midbrain	Abbreviations	Label
Cortico-spinal tract	8 (16%)	12 (24%)	6 (12%)	CST	1
Medial lemniscus	7 (14%)	10 (20%)	7 (14%)	ML	2
Cuneate and gracile fasciculi	5 (10%)			CFGF	2.1
Internal arcuate fibers	5 (10%)			IAF	2.2
Spino thalamic tract	9 (18%)	22 (44%)	11 (22%)	STT	3
Medial longitudinal fasciculus	4 (8%)	17 (34%)	9 (18%)	MLF	4
Transverse fibers of the pons		33 (66%)		TFP	5
Spino cerebellar tract	6 (12%)			SCT	6
Central tegmental tract	3 (6%)	9 (18%)	8 (16%)	CTT	7
Fronto pontine fibers			2 (4%)	FPF	8
Parieto- occipito- temporo pontine fibers			3 (6%)	POTPF	9
Lateral lemniscus		18 (36%)	6 (12%)	LL	10
Inferior cerebellar peduncle	4 (8%)			ICP	11
Middle cerebellar peduncle		14 (28%)		MCP	12
Superior cerebellar peduncle		39 (78%)	3 (6%)	SCP	13
Hypoglossal nucleus and nerve	14 (28%)			XII	14
Solitary nucleus and tract	20 (40%)			ST	15
Vague nerve	1 (2%)			X	15.1
Vestibular and cochlear nuclei and nerves	16 (32%)	17 (34%)		VIII	16
Facial nucleus and nerve		20 (40%)		VII	17
Abducens nucleus and nerve		17 (34%)		VI	18
Trigeminal tract	12 (24%)	15 (30%)	3 (6%)	V	19
Oculomotor nucleus and nerve			7 (14%)	III	20
Olivary complex	16 (32%)			OC	21
Red nucleus			3 (6%)	RN	22
Substantia nigra			6 (12%)	SN	23
Inferior colliculus			1 (2%)	IC	24
Superior colliculus			1 (2%)	SC	25
Periventricular gray	5 (10%)	20 (40%)	1 (2%)	PVG	26
Periaqueductal gray			14 (28%)	PAG	27
Raphe nuclei		13 (26%)	7 (14%)	R	28
Reticular formation	2 (4%)	6 (12%)	4 (8%)	RF

### Medulla oblongata

The distinctive signal of tracts and nuclei in FGATIR allowed identifying hypersignals in small structures of the medulla oblongata. At the spinal cord—medulla junction, a hypersignal was observed on ventral paramedial CST pyramid in 8 patients *(16%)* (1.1, B3,B5). A dorsolateral hypersignal was observed on the V tract in 12 patients *(24%),* sometimes at several levels of the medulla oblongata (19, B1,B2). Hypersignals might be limited (ST 15, B4) or extended to several structures (CFGF 2.1, IAF 2.2, SCT 6, V 19, B2).

At an upper level, a periventricular hypersignal frequently encompassed from medial to lateral, the XII nucleus *(28%),* ST *(40%)* and the VIII nuclei *(32%),* with ICP as a lateral border at the vague nerve level (11, 14, 15, 15.1, 16, B7-B9). In ventral area, involvement of OC was observed in 16 patients *(32%)* (21, B6,B7). MS hypersignals sometimes extended beyond OC to CTT, and reached STT, SCT and V dorsally, ML medially (2, 3, 6, 7, 19, B6,B9,B10). ICP was only affected in 4 patients *(8%)* with a significant hypersignal compared to contralateral (11, B9).

### Pons

- Tegmentum pontis (P1–P5):

The VI nerve was mostly affected at its nucleus in paramedial periventricular area, but also on its pathway through the basis pontis, with a linear hypersignal along the nerve *(34%)* (18, P2). Paramedial ML inflammation displayed a hypersignal contrasting with the contralateral normal hypointensity in 10 patients *(20%)* (2, P2).

Above the VI nucleus level, the inflammatory periventricular gray (PVG) highlighted in 20 patients a sharp « nail stroke» hypersignal distinctive from the apparently spared paramedial reticular formation *(40%).* The periventricular hypersignal frequently extended to SCP at the lateral wall of the 4th ventricle (13, 26, P3-P5). Of note, in our cohort, SCP was the most affected structure, concerning nearly 4 patients out of 5 *(78%).* In 13 patients, a midline hypersignal concerned the raphe nuclei (R) with a well defined triangular shape. Unilateral inflammation of MLF adjacent to R nuclei highlighted a typical one-eyed feature at the midline instead of the normal double dot on axial sections (4, 28, P2,P3,P5).

At the medulla—pons junction, a lateral oblique hypersignal involved the VII nucleus and nerve throughout the basis pontis *(40%),* as well as the superior vestibular nucleus (SVN) bulging at the lateral wall of the 4^th^ ventricle *(34%)* (16, 17, P1-P4). In the rostral pons, a lateral oblique hypersignal affected the motor and the principal nuclei of the V nerve in 15 patients *(30%),* frequenly extending to the exiting nerve (19, P4).

- Basis pontis:

Throughout the pons, the transversal fibers of the pons (TFP) were affected in two thirds of cases, with an elective anterolateral and/or anteromedial hypersignal (5, P2,P3). In paramedial areas, hypersignals were less frequently denoted within the crossing fibers of CST and TFP *(24%)* (1, P4). MCP connecting TFP to the cerebellum was affected in 14 patients *(28%)* (12, P2).

### Midbrain

In the tegmentum, PAG was frequently affected *(28%)* (27, M4, M5), with large hypersignals sometimes overflowing to paramedial RF *(8%).* The small trochlear nucleus was uneasy to delineate at the inferior colliculus level. At the superior colliculus level, hypersignals of the III nerve were denoted in 7 patients *(14%),* involving the nucleus and/or the nerve along its way through the red nucleus (20, M2,M4). Medial lesions were found on MLF tracts and discernable throughout PAG hypersignal *(18%).* Lateral involvement of the tegmentum was found on STT *(22%)* with neighboring ML *(14%),* and LL *(12%)* (2,3,10, M1,M2,M4). SCP appeared less affected at the midbrain decussation level *(6%).* Hypersignals were also rarely found in the RN *(6%)* (22, M5) and in the SN *(12%)* (23, M2).

In the crus cerebri, hyperintensities were mostly observed in CST *(12%)* (1, M1), FPF *(4%)* and POTPF *(4%)* (9, M3). In the tectum, large PAG hypersignals encompassed the inferior and superior colliculi in 2 patients (25, M4). In one patient, lesional hypersignals extended to the right medial geniculate via the brachium of the inferior colliculus (24, M5).

At the three levels of the brainstem, MS lesions of the reticular formation (RF) remained difficult to identify. Lateral RF was possibly included in the hyperintensity covering the V, VI, VII and VIII nerves, while paramedial RF mostly appeared spared. In the pons *(26%)* and the midbrain *(14%),* the affected raphe nuclei (R) corresponded to a medial linear hyperintensity running through the tegmentum pontis (28, M3).

### MS hypersignals assessment and Inter-observer agreement

ROIs were drawn in 24 patients, including 8 measurements in V tracts, 9 measurements in CST pyramids, and 17 measurements in MLF tracts. One can note concomitant involvements of different structures in a same patient. The mean CR of CRLesion and CRContralateral were 11.7 ± 7.4 and 14.5 ± 8.6, respectively. The mean CR of CRLesion was significantly lower than the mean CR of CRContralateral (*p* < 0.01).

**Fig. 6:**
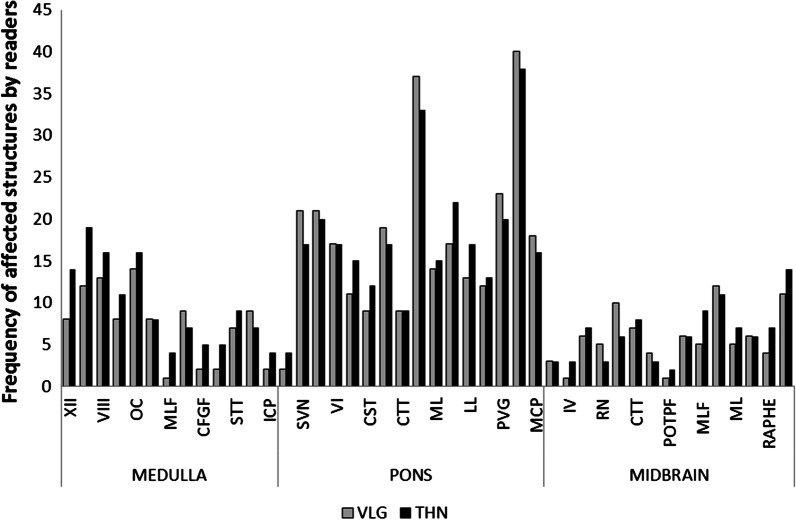
Interobserver evaluation of brainstem structures affected by MS

Spatial resolution of PD/T2w and signal differentiation of FGATIR offered a precise and reproducible localization of pathological hypersignals in the brainstem, confirmed with an overall Cohen’s kappa score of 0.71, corresponding to a substantial interobserver agreement (Fig. 6).

With a respective Cohen’s kappa score of 0.61, 0.76, 0.62 for medulla, pons and midbrain, the maximum interobserver agreement was found in the voluminous pons, with a frequent involvement of TFP and SCP, and a comparable detection rate in tracts and nuclei. Nuclei appeared more discernable than tracts in the medulla, and inversely in the midbrain.

## Discussion

Our study showed that the FGATIR sequence allows a direct anatomical location of inflammatory lesions identified by PD/T2w within the brainstem, more precisely than indirect extrapolations from histological sections. Moreover, we observed in our MS patients an elective involvement of some anatomical structures. Hypersignals were more frequently observed in the voluminous pons than in the medulla oblongata and the midbrain. As for the supratentorial region, the periventricular gray was confirmed as an elective site of MS inflammation throughout the brainstem. TFP and SCP appeared as the most frequently affected pathways, but smaller tracts such as STT, ML and MLF and little structures of the medulla oblongata were also clearly identified with the signal discrimination of FGATIR and the spatial resolution of PD/T2w sequences. Large hypersignals often encompassed nuclear groups such as XII, ST, and VIII in the medulla, V, VI and VII in the pons, but with different signal features.

As an essential structure joining the telencephalon to the spinal cord, the brainstem is a prime target of MS disease due to the high density of internal structures. Nevertheless, exploration of the brainstem remains difficult due to the complex organization of tracts and nuclei within a restricted volume. While the inflammatory lesion distribution has been well described in the telencephalon, only few studies brought out direct disclosure of the brainstem intrinsic anomalies, often combining neurophysiological and radiological explorations to elucidate the clinical complain [28–32].

Recent developments in MRI techniques focus on signal discrimination of the brainstem components, to allow segmentation and volumetric measurement of brainstem regions in a long term follow-up of degenerative diseases [8, 33]. In our study, we have used the signal discrimination offered by the FGATIR sequence to identify the brainstem structures electively concerned by the inflammatory process, in order to elaborate an anatomical template of brainstem injury in MS.

Previous studies already assessed MS hypersignal detection in the infratentorial area using 2D PD/T2w and 3D FLAIR [34–36]. Firstly considered as less sensitive in the posteria fossa, 2D and 3D FLAIR sequences are now challenging PD/T2w sequence regarding brainstem signal discrimination. However, it is noteworthy that the PD/T2w sequence achieves a higher spatial resolution than FLAIR imaging with a comparable sequence duration. In our study, this spatial resolution offered a precise delineation of the pathological hypersignal. Indeed, the sharply defined hypersignal of periventricular gray matter differs from the blurry hypersignal in lateral pons, and raises questions concerning the mecanism of myelin inflammation [37–40]. Such variable hypersignal features suggest a specific interaction of the glial and vascular infrastructures in the inflammatory process, at each area of the brainstem [19, 41].

Our preliminary study had several limitations. First, our cohort only included 50 patients with a heterogeneous population due to a large age ranking. A reliable statistical evaluation of MR imaging semiology would require a larger patient population and an initial statement of MS grading, clinical description and informations about treatments. However, the goal of this preliminary study was only to demonstrate that a combination of PD/T2w and a dedicated 3D FGATIR sequence could help to precisely delineate the brainstem structures affected by MS. Further investigation should be performed with a prospective enrollment of MS patients with assessed clinical condition in order to statistically validate diagnostic correlation through FGATIR and PD/T2w.

The long scan time of our FGATIR sequence was mandatory to provide a submillimetric spatial resolution and a high signal contrast of the brainstem structures. It may as well induce image quality corruption by motion artefacts in uncooperant patients. Secondly, the acquisition was limited to the infratentorial area, and should be extended to the whole brain for MS assessment in clinical routine. Thirdly, gradients in hypersignals for normal periventricular gray and nuclei through FGATIR may not be easily distinguished from an abnormal hypersignal, thus requiring the signal contrast and spatial resolution of PD/T2w to assess a real signal anomaly. For this reason, the use of FGATIR sequence is limited in clinical routine and should be useful only when associated to conventional sequences. These concerns may be addressed with most recent techniques that dramatically reduce the number of phase encoding step needed to complete the k-space, including compressed sensing image reconstruction, Wave-controlled aliasing in parallel imaging or deep learning approaches [42–45].

Further longitudinal exploration of MS lesional history would benefit from tractography method which allows investigation of the temporal evolution of the fiber anisotropy and orientation within an inflammatory plaque [23]. Moreover, diffusion tractography may provide complementary contrast to FGATIR for a better identification of small dimension structures (i.e. the IV nucleus) [41, 46]. Finally, additional information based on relaxometry technique would bring an accurate information about MS lesion quantification in FGATIR, to define a cut-off value in regard to normal hypersignals for an automatic lesion segmentation of the brainstem [37].

These approaches would be perfectly in phase with the recent technical advances reported in literature, indicating significant potential in the field of quantitative brain MR image analysis. Indeed, the important development of artificial intelligence techniques have had a major impact on medical imaging study of MS, with a focus on disease classification, detection/segmentation or predicting the course of disease [47]. Deep learning-based approaches for brain MRI are gaining interest due to their self-learning and generalization ability over large amounts of data [45]. A variety of methods have been developed and applied in the context of MS, including identification of multiple sclerosis subtypes or automatic lesions segmentations [48–50].

In this study, we emphasized the opportunity to precisely assess the anatomical structures of the brainstem affected by MS based on FGATIR sequence. These supplementary information could be used for classification purpose to further characterize the long term evolution of the MS disease in patient follow up. This may help to improve patients outcoming by facilitating the earlier diagnosis and prediction of MS evolution.

## Conclusion

The high signal discrimination in FGATIR offered a macroscopic identification of brainstem macroscopic anatomy while combination with PD/T2w opened prospects to define MS elective injury in tracts and nuclei. In addition to the frequent involvement of tracts running through the brainstem, the inflammatory hypersignal also concerns the nuclear groups and the reticular formation as well. Our results suggest as a complement to the evaluation of the telencephalon, a specific semeiology of the brainstem injury in MS, combining clinical, radiological and neurophysiological imputs. In addition to the development of an anatomical template orienting the machine learning as part of the longitudinal monitoring of MS, this first radioclinical approach should help to estimate the long-term clinical impact of MS in specific structures of the brainstem.

## Data Availability

The datasets used and/or analysed during the current study are available from the corresponding author (T.H. Nguyen) on reasonable request.

## Abbreviations

CFGF: Cuneate and gracile fasciculi
CST: Cortico-spinal tract
CTT: Central tegmental tract
FPF: Frontopontine fibers
IAF: Internal arcuate fibers
IC: Inferior colliculus
ICP: Inferior cerebellar peduncle
III: Oculomotor nucleus and nerve
LL: Lateral lemniscus
MCP: Middle cerebellar peduncle
ML: Medial lemniscus
MLF: Medial longitudinal fasciculus
OC: Olivary complex
PAG: Periaqueductal gray
POTPF: Parieto-, occipito-, temporo pontine fibers
PVG: Periventricular gray
R: Raphe nuclei
RF: Reticular formation
RN: Red nucleus
SC: Superior colliculus
SCP: Superior cerebellar peduncle
SCT: Spino cerebellar tract
SN: Substantia nigra
ST: Solitary nucleus and tract
STT: Spino thalamic tract
TFP: Transverse fibers of the pons
V: Trigeminal tract
VI: Abducens nucleus and nerve
VII: Facial nucleus and nerve
VIII: Vestibular and cochlear nuclei and nerves
X: Vague nerve
XII: Hypoglossal nucleus and nerve
